# An attacin antimicrobial peptide, Hill_BB_C10074, from *Hermetia illucens* with anti-*Pseudomonas aeruginosa* activity

**DOI:** 10.1186/s12866-023-03131-1

**Published:** 2023-12-01

**Authors:** Leila Fahmy, Youssif M. Ali, David Seilly, Reece McCoy, Róisín M. Owens, Miha Pipan, Graham Christie, Andrew J Grant

**Affiliations:** 1 Department of Veterinary Medicine, University of Cambridge, Cambridge, UK; 2 Department of Chemical Engineering and Biotechnology, University of Cambridge, Cambridge, UK; 3 Better Origin, Future Business Centre, Cambridge, UK

**Keywords:** Alphafold, Antimicrobial peptides, Antibiotics, Attacins, *Hermetia illucens*, Membrane permeability, *Pseudomonas aeruginosa*

## Abstract

**Background:**

There is a global need to develop new therapies to treat infectious diseases and tackle the rise in antimicrobial resistance. To date, the larvae of the Black Solider Fly, *Hermetia illucens*, have the largest repertoire of antimicrobial peptides derived from insects. Antimicrobial peptides are of particular interest in the exploration of alternative antimicrobials due to their potent action and reduced propensity to induce resistance compared with more traditional antibiotics.

**Results:**

The predicted attacin from *H. illucens*, Hill_BB_C10074, was first identified in the transcriptome of *H. illucens* populations that had been fed a plant-oil based diet. In this study, recombinant Hill_BB_C10074 (500 µg/mL), was found to possess potent antimicrobial activity against the serious Gram-negative pathogen, *Pseudomonas aeruginosa*. Sequence and structural homology modelling predicted that Hill_BB_C10074 formed a homotrimeric complex that may form pores in the Gram-negative bacterial outer membrane. In vitro experiments defined the antimicrobial action of Hill_BB_C10074 against *P. aeruginosa* and transmission electron microscopy and electrochemical impedance spectroscopy confirmed the outer membrane disruptive power of Hill_BB_C10074 which was greater than the clinically relevant antibiotic, polymyxin B.

**Conclusions:**

Combining predictive tools with in vitro approaches, we have characterised Hill_BB_C10074 as an important insect antimicrobial peptide and promising candidate for the future development of clinical antimicrobials.

**Supplementary Information:**

The online version contains supplementary material available at 10.1186/s12866-023-03131-1.

## Background

Antimicrobial resistance (AMR) is a global public health concern that is driving the urgent need to develop new therapies to treat infectious diseases. In 2019, an estimated 4.95 million deaths were associated with bacterial AMR [1] and since the outbreak of the COVID-19 pandemic, co-infections of SARS-CoV-2 and bacterial pneumonia has seen a rise in the rate of AMR development [2]. To meet the challenge of AMR, research into less conventional sources of therapeutics such as antimicrobial peptides (AMPs) is intensifying.

AMPs exist widely in nature as important effectors of innate immune systems against microbial invasions [3]. AMPs are primarily characterised as small proteins typically formed from fewer than 100 amino acids and under 10 kDa [3]. They are often rich in positively charged and hydrophobic amino acid residues conferring an amphiphilic structure that facilitates interactions with negatively charged surfaces such as some membranes [3]. Due to their physicochemical make-up AMPs demonstrate a broad-spectrum of antimicrobial activities against various microorganisms including pathogenic bacteria, fungi and viruses [3]. The mechanisms of the antimicrobial action of AMPs are highly diverse with the ability of AMPs to disrupt membranes being the most explored [4]. However, AMPs also target other components of the cell wall as well as intracellular components. Furthermore, AMPs can serve multiple functions and as such interact with several membrane and non-membrane-based targets in different bacterial species [5]. Certain AMPs may kill one species of bacteria through membrane disruption whilst against another species they may target intracellular structures instead [6]. This display of multifunctionality indicates that the antimicrobial action of AMPs may not be the primordial function of the proteins. At the time of writing, over 8,000 AMP sequences have been deposited on the Collection of Antimicrobial Peptides R3 database (CAMPR3) [6].

A large proportion of AMPs were first identified in insects including cecropins, ponericins, defensins, lebocins, drosocin, metchnikowin, gloverins, diptericins and attatcins [7]. Insects rely solely on innate immune system mechanisms making them a rich source of AMPs whose expression is controlled through the TOLL and Immune Deficiency pathway [8]. The greater wax moth, *Galleria mellonella*, and the fruit fly, *Drosophila melanogaster*, are organisms that are traditionally studied for insect immune responses. However, recently, the immune system of the Black Soldier Fly, *Hermetia illucens* (L.) (Diptera: Stratiomyidae), has attracted attention [9]. With the ability to thrive in microbially rich environments, populations of *H. illucens* larvae are thought to be rich with AMPs [10]. To date, 14 AMPs from *H. illucens* have been characterised for their in vitro antimicrobial activity against bacteria [11–19]. *H. illucens* AMPs have been identified as members of the defensins, attacin and diptericin families [20]. In addition to these characterised AMPs, many other candidate sequences were predicted from the species to possess antimicrobial functionality [21, 22].

More than 50 AMP sequences were predicted from the transcriptome of *H. illucens* in response to dietary changes [21]. Following exposure to a plant oil-based diet, *H. illucens* larvae expressed a 148-residue protein which was predicted to be an attacin and was named Hill_BB_C10074. The attacin family of AMPs were first identified from the haemolymph of *Hyalophora cecropia* [21], and have since been isolated from several other insects [23]. Attacins are relatively large AMPs (~ 20 kDa) and are rich in glycine residues and deficient in cysteines. Due to this biochemical makeup, attacins are thought to adopt a random coil structure in aqueous solutions but their secondary structures in membrane environments have not yet been solved [23]. At the time of writing, the predicted structures of the 24 *D. melanogaster* attacins or attacin-like AMPs deposited on the UniProt database contain β-sheet elements (accessed: 13/02/2023) [24]. They are expressed as pre-pro-peptides and undergo proteolytic processing to form a mature peptide which contains between 14 and 22% glycine residues. Mature attacins display a net overall charge between − 3 to + 10, due to this large range, the charged nature of attacins may not be the most crucial characteristic to underpin the family’s mechanism of antimicrobial activity. Attacins have specific antimicrobial activity against Gram-negative bacteria and so provide a promising candidate for treatment of some of the most concerning bacterial infections [25].

Building on the preliminary predictive work on Hill_BB_C10074 [21], we used a combination of *in silico* and in vitro approaches to produce and characterise the AMP. The mature Hill_BB_C10074, produced as a recombinant 6xHis-tagged protein, was found to possess anti-bacterial activity against the serious Gram-negative bacterial pathogen *Pseudomonas aeruginosa*. Hill_BB_C10074 was proposed to behave similarly to a homotrimeric porin and it was shown to disrupt the Gram-negative bacterial outer membrane.

## Results

### *In silico* characterisation of the putative attacin, Hill_BB_C10074

**Fig. 1:**
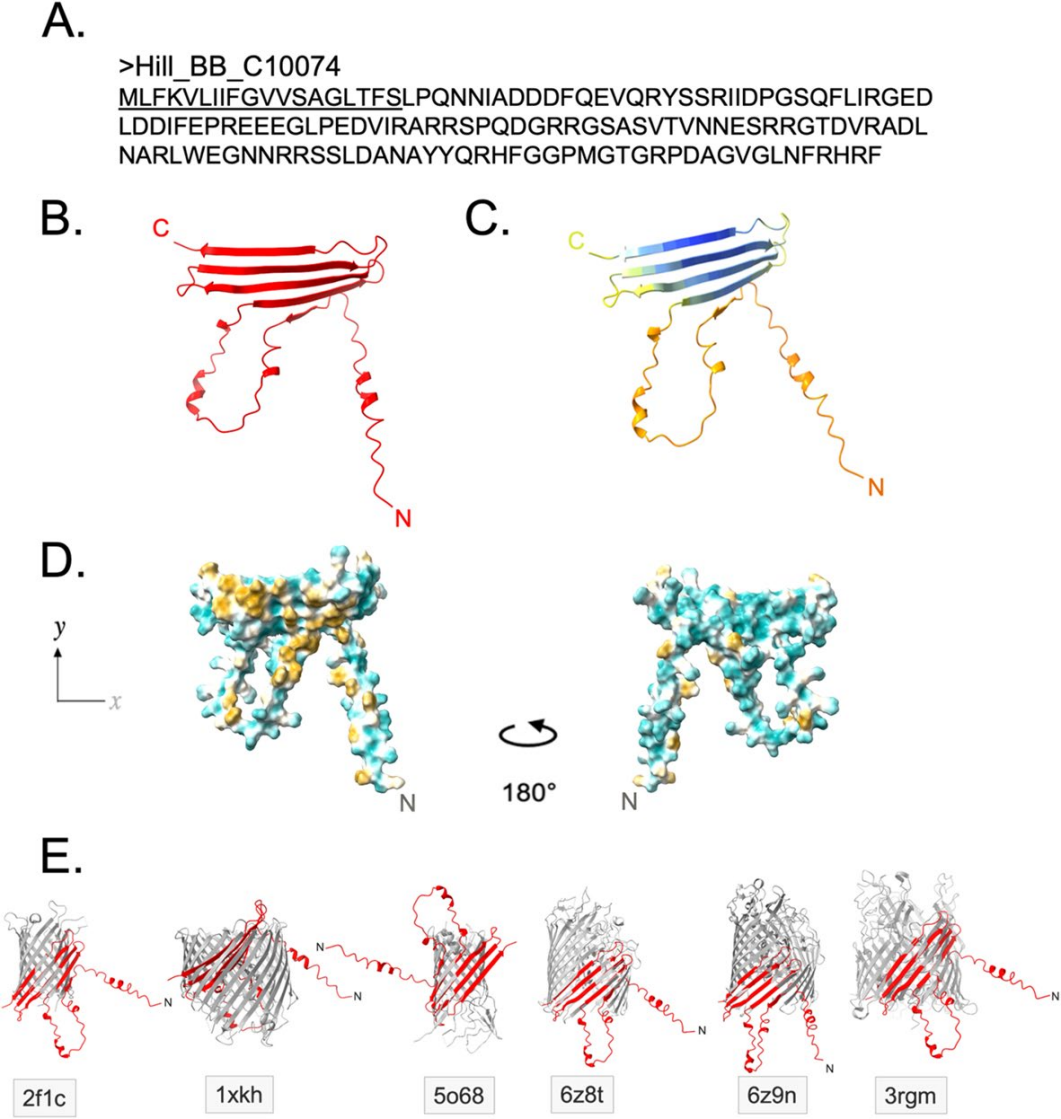
AF2 prediction of Hill_BB_C10074 monomer. **A **Amino acid sequence of Hill_BB_C10074. Signal peptide underlined. **B **AF2 prediction of mature Hill_BB_C10074. **C **AF2 model prediction confidence of Hill_BB_C10074. **D **Hydrophobicities of mature Hill_BB_C10074 with highly hydrophilic regions coloured in cyan and regions highly hydrophobic coloured in gold. **E **Structural overlays of mature Hill_BB_C10074 model and similar protein structures. PDB identity of each protein detailed in grey boxes

A 20-residue N-terminal signal peptide was removed from Hill_BB_C10074 to form a 130 amino acid sequence encoding a 14.8 kDa mature protein (Fig. 1A). Typical of attacins, Hill_BB_C10074, lacked cysteines, was abundant with charged residues and had a high glycine composition. AlphaFold v2.1.0 (AF2) modelled Hill_BB_C10074 with a disordered N-terminus containing 3 short α-helices and a β-sheet C-terminus formed of 5 antiparallel strands (Fig. 1B). Confidence in the AF2 predicted β-sheet region of the model was good (Fig. 1C). The structure presented with amphipathicity and contained hydrophilic and hydrophobic faces (Fig. 1D).

To date, no tertiary structures of attacins have been experimentally solved (PDB search performed July 2023). Despite this, the AF2 model of Hill_BB_C10074 shared structural similarity with 1,917 experimentally solved protein structures deposited on the PDB (search performed July 2023). The top 10 proteins with similar structures to Hill_BB_C10074 all formed membrane pores and 7 out of the 10 were specific to Gram-negative bacterial membranes (Table 1). Structure-Activity-Relationship (SAR) analysis of these data infer that Hill_BB_C10074 possesses membrane pore forming functionality with possible specificity towards Gram-negative bacterial membranes.

**Table 1:** Proteins sharing structural folds with the putative attacin, Hill_BB_C10074

PDB entry-Chain	Z score	rmsd	%id	PDB Description	Source organism	Stoichiometry	Membrane protein?
**6h04-I**	7.2	23.2	3	Complement component C9	*Homo sapiens*	Hetero 24-mer	Yes
**5fq6-M**	6.5	15.6	11	Putative lipoprotein	*Bacteroides thetaiotaomicron*	Hetero 8-mer	Yes
**5dl5-A**	6.3	11.5	8	Membrane protein	*Acinetobacter baummanii*	Monomer	Yes
**4pr7-A**	6.2	2.9	11	Oligogalacturonate-specific porin	*Dickeya dadantii*	Monomer	Yes
**6zlt-B**	6.2	14.6	10	SusD homolog	*B. thetaiotaomicron*	Hetero 4-mer	Yes
**4c00-A**	6.1	11.8	8	Translocation and assembly module	*E. coli*	Monomer	Yes
**2gsk-A**	6.1	10.1	11	Vitamin B12 transporter BtuB	*E. coli*	Hetero 2-mer	Yes
**4epa-A**	6.1	13.4	12	Pesitcin receptor	*Yersinia pestis*	Monomer	Yes
**1wzn-A**	5.8	12.2	7	Sam-dependent methyltransferase	*Pyrococcus Hiroshii*	Homo 6-mer	Unknown
**2w4y-A**	5.8	4.6	3	Caulobacter 5 virus-like particle	Unclassified *Emesvirus*	Homo 180-mer	No

The β-sheet of Hill_BB_C10074 shared structural similarity with the transmembrane β-barrel structures in the 10 most similar protein structures listed in Table 1. Superimposition of Hill_BB_C10074 against the membrane proteins (Fig. 1E) indicated that stoichiometry of the attacin may be a monomeric subunit of a multimeric protein.

**Fig. 2:**
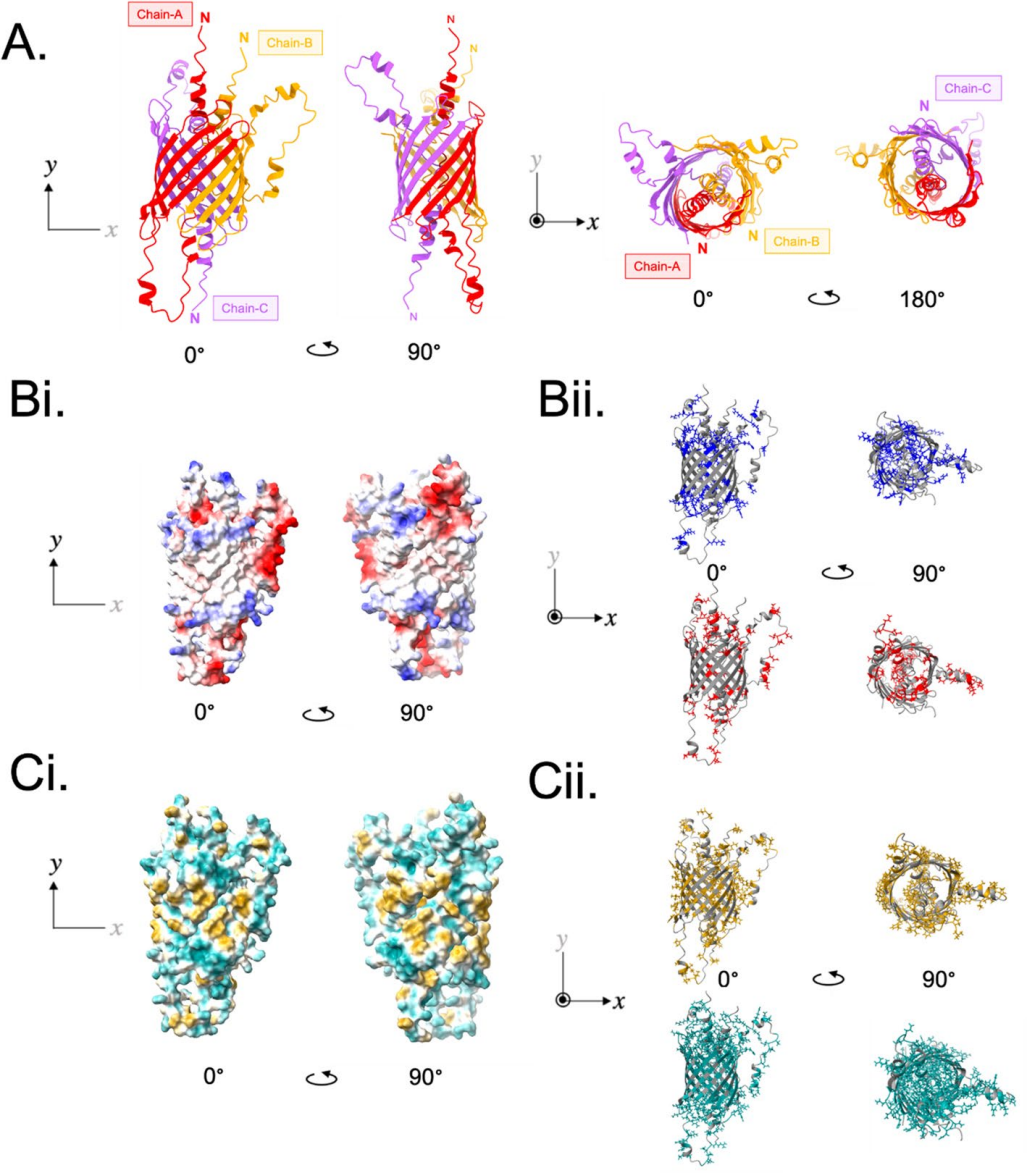
AF2 prediction of Hill_BB_C10074 homotrimer. **A **View of the Hill_BB_C10074 homotrimeric model through *x* and *y* axes. The 3 chains of the Hill_BB_C10074 homotrimer are coloured red (Hill_BB_C10074-A), orange (Hill_BB_C10074-B) and purple (Hill_BB_C10074-C). **B **Electrostatic surface of the homotrimer prediction with positively charged amino acid residues coloured blue, and negatively charged residues coloured red. **C **Hydrophobic surface of the Hill_BB_C10074 homotrimer prediction with highly hydrophilic residues coloured in cyan, and highly hydrophobic residues coloured in gold

Membrane porins are frequently composed of 3 oligomers that form a β-barrel channel [26]. AF2 was used to predict the homotrimeric structure of Hill_BB_C10074 (Fig. 2A). The homotrimeric model of Hill_BB_C10074 revealed a β-barrel structure formed of 12-antiparallel strands, which is characteristic of membrane porins [26]. Two of the homotrimer chains had the same orientations with both N-terminal loops feeding through the β-barrel *via* one aperture of the structure, whilst the third chain was orientated inversely, with its N-terminal loop fed through the β-barrel through the opposing end of the β-barrel’s lumen.

The electrostatic surface of the Hill_BB_C10074 homotrimer revealed charged regions gathered near the poles of the β-barrel and within the lumen of the structure (Fig. 2Bi). These charged regions corresponded to the N-terminal loops whilst the β-sheets that formed the barrel structure were predominantly neutral. Acidic residues were contained on the N-terminal loops and thread through the lumen of the β-barrel, whilst basic residues clustered around entrances of the β-barrel, creating spaces of dense positive charge (Fig. 2Bii).

Mapping of the hydrophobic and polar residues of the Hill_BB_C10074 homotrimer was typical of pore forming β-barrel proteins which are composed of a hydrophobic external surface encasing a polar internal core (Fig. 2Ci). This hydrophobic surface facilitates the proteins’ interaction with polar headgroups of lipopolysaccharide molecules on the Gram-negative bacterial outer membrane and embeds the protein within the lipid milieu of a membrane bilayer [27]. Whilst polar amino acids project into the internal lumen of the β-barrel creating an aqueous pore [27]. The hydrophobic and polar residues of the Hill_BB_C10074 homotrimer were arranged through dyad symmetry, allowing the hydrophobic residues’ R-side chains to project onto the surface of the protein and the polar residues’ R-side chains to project into the lumen of the β-barrel (Fig. 2Cii).

### Recombinant production of the putative attacin, Hill_BB_C10074

**Fig. 3:**
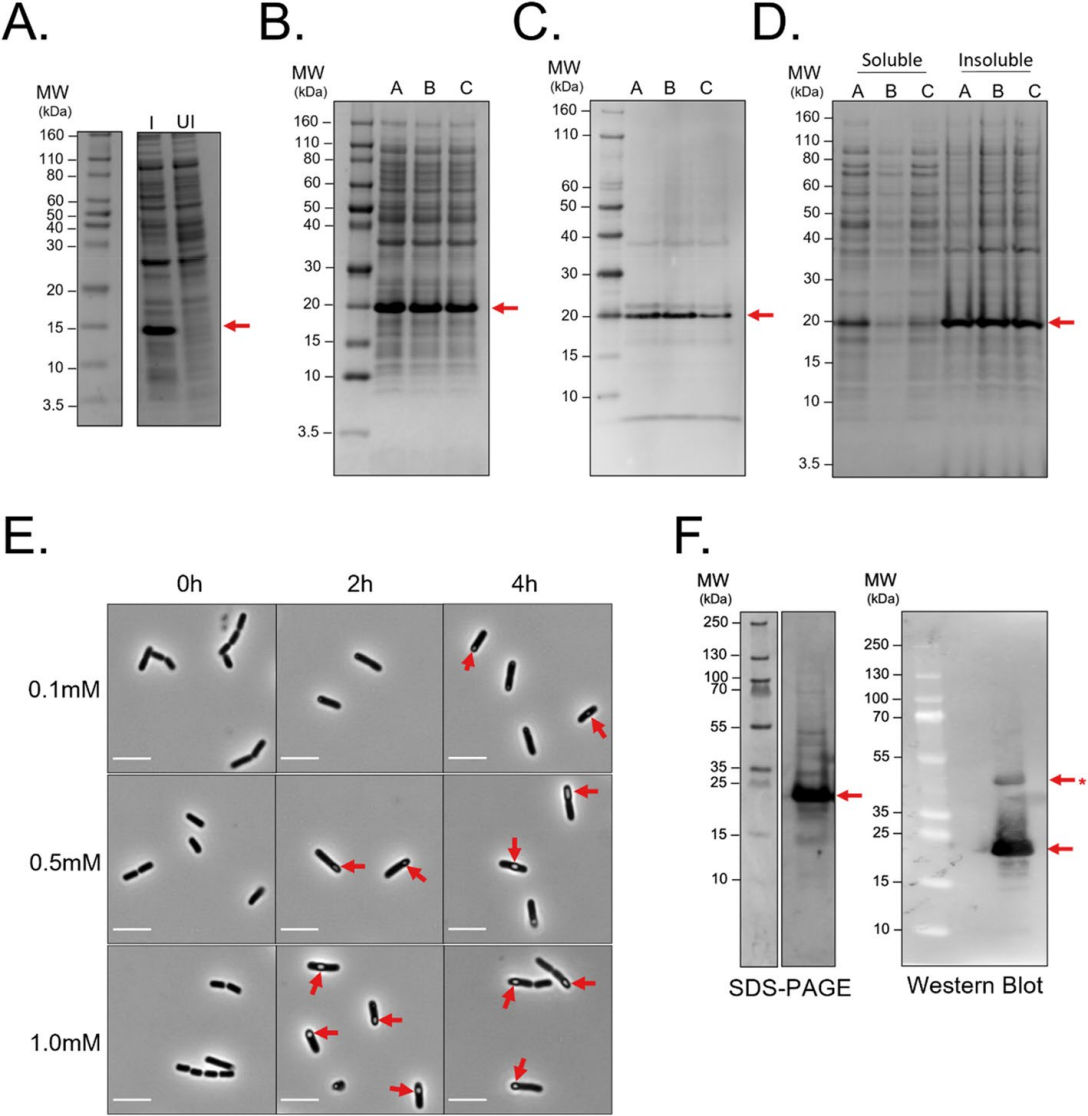
Recombinant protein expression of 6xHis-Hill_BB_C10074. **A **SDS-PAGE of whole cell lysate from *E. coli* BL21 (DE3) cells transformed with T7 inducible plasmid encoding the 6xHis-Hill_BB_C10074 constructs induced (I) with 1.0 mM IPTG and uninduced (UI). **B **SDS-PAGE of total protein from *E. coli* BL21(DE3) cells induced with either 1.0 mM (**A**), 0.5 mM (**B**) or 0.1 mM (**C**) IPTG for 4 h. (**C**) Western blot using anti-6xHis antibody to detect 6xHis-Hill_BB_C10074 following expression in E. coli BL21(DE3) cells induced with either 1.0 mM (A), 0.5 mM (B) or 0.1 mM (C) IPTG for 4-hours. **D **SDS-PAGE of solubility fractionated total-cell protein contents following expression induced with either 1.0 mM (**A**), 0.5 mM (**B**) or 0.1 mM (**C**) IPTG for 4-hours to determine whether 6xHis-Hill_BB_C10074 was soluble or insoluble. **E **Phase-contrast visualisation of E. coli BL21(DE3) cells expressing 6xHis-Hill_BB_C10074 into inclusion bodies over the course of 4 h of induction in response to varying concentrations of IPTG (0.1 mM, 0.5 mM and 1.0 mM). Scale bar = 5 µM. **F **SDS-PAGE and western blot of purified 6xHis-Hill_BB_C10074. A higher MW band (indicated with an asterisk) may be a multimer of 6xHis-Hill_BB_C10074. In all images, recombinant 6xHis-Hill_BB_C10074 protein is indicated by a red arrow. Full length gels and blots are shown in Supplementary Fig. 1

Recombinant 6xHis-Hill_BB_C10074 was expressed in *Escherichia coli* BL21(DE3). The presence of 6xHis-Hill_BB_C10074 in the bacterial whole cell lysate was evaluated through SDS-PAGE (Fig. 3A and B) and the 6xHis tag was detected through western blotting (Fig. 3C). Recombinant 6xHis-Hill_BB_C10074 was expressed in the insoluble fraction of the whole cell lysate, irrespective of IPTG concentration used to induce expression (Fig. 3D). Large inclusion bodies were identified in cells overexpressing 6xHis-Hill_BB_C10074 (Fig. 3E). Recombinant 6xHis-Hill_BB_C10074 was isolated from bacterial cultures that had been induced with 0.5mM IPTG. Purification strategies focused on the solubilisation of recombinant protein followed by isolation through nickel affinity chromatography and refolding of 6xHis-Hill_BB_C10074. SDS-PAGE and western blotting of the purified protein detected a band between the 15 kDa – 25 kDa markers which was consistent with the predicted MW of the 6xHis-Hill_BB_C10074 monomer (18.3 kDa) (Fig. 3F). A second band, roughly double the MW of 6xHis-Hill_BB_C10074, was also present in the western blot sample. This suggested that even under reducing conditions, 6xHis-Hill_BB_C10074 may form multimers.

### Antimicrobial activity assay of 6xHis-Hill_BB_C10074

**Fig. 4:**
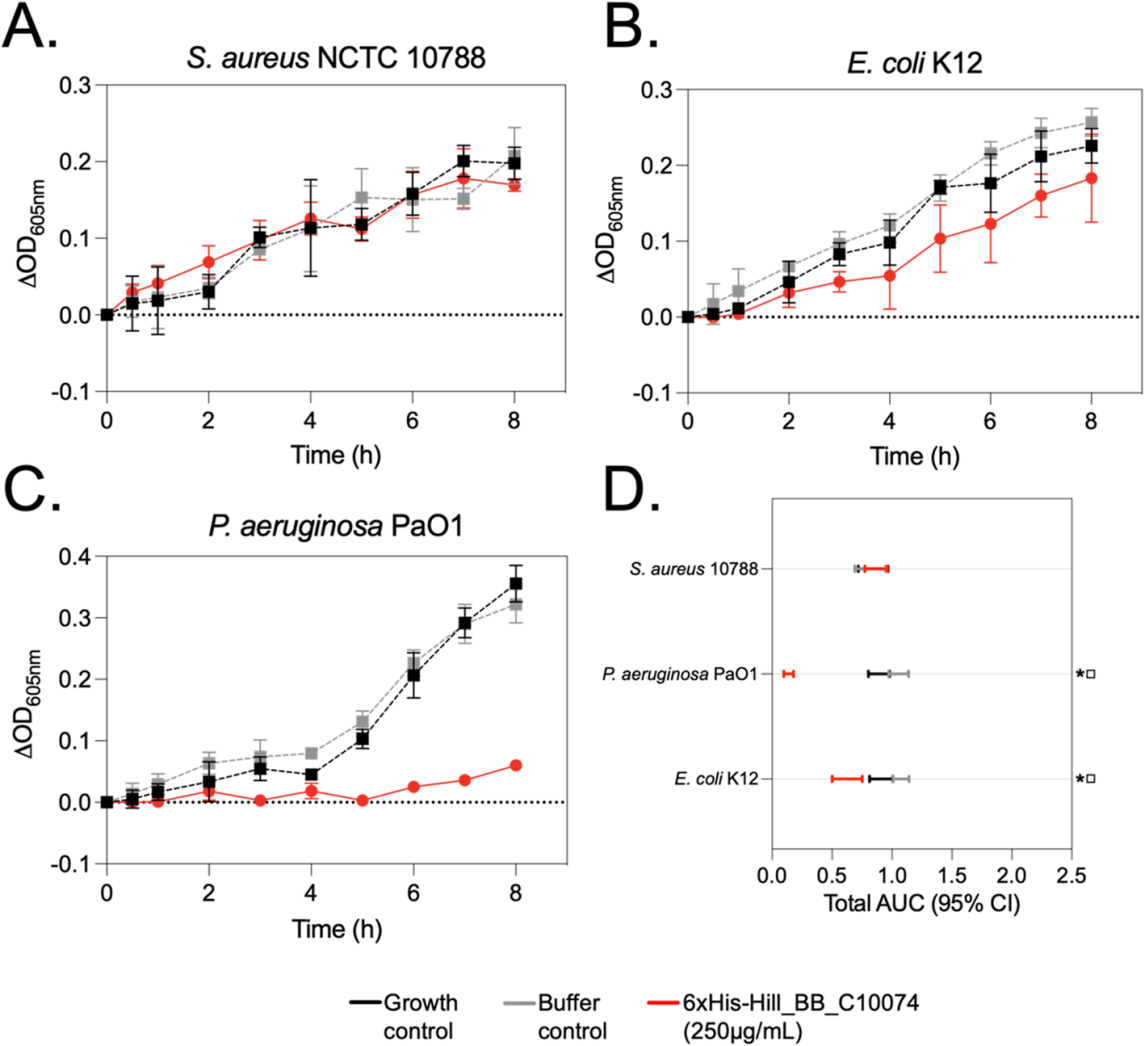
Optical Density measurements of 3 species of bacteria exposed to 6xHis-Hill_BB_C10074. **A ***S. aureus* NCTC 10,788, **B ***E. coli* K12, and **C ***P. aeruginosa* PaO1 were grown in 200 µL wells containing Hill_BB_C10074 (250 µg/mL) for 8-hours. OD_605nm_ of the cultures were measured periodically and changes in absorbance indicated alterations in growth of the populations. Growth and Buffer (25 mM TrisHCl pH 8.0) controls were included. Data is representative of 3 independent experiments. Error bars represent standard deviations of the mean. **D **AUCs for each of the growth curves were calculated. Error bars represent 95% CI. Symbols denote significant reduction in AUC between populations. * = 6xHis-Hill_BB_C10074 (250 µg/mL) population reduced compared to the Growth control population. □ = 6xHis-Hill_BB_C10074 (250 µg/mL) population reduced compared to Buffer control population. Populations labelled with the combination of *□ were reduced in size as a direct effect of 6xHis-Hill_BB_C10074 (250 µg/mL)

6xHis-Hill_BB_C10074 was initially screened using in vitro phenotypic assays for antimicrobial activity against the Gram-positive species *Staphylococcus aureus* NCTC 10,788, and the Gram-negative bacterial species *E. coli* K12 and *Pseudomonas aeruginosa* PaO1. In a 96-well plate format, bacterial cultures were incubated with recombinant 6xHis-Hill_BB_C10074 (250 µg/mL) for 8-hours, and ODs were measured periodically to monitor the effect of the purified protein. Under test conditions, 6xHis-Hill_BB_C10074 had no measurable antimicrobial activity against *S. aureus* NCTC 10,788 (Fig. 4A). However, 6xHis-Hill_BB_C10074 (250 µg/mL) did inhibit the growth of the Gram-negative species tested. The inhibitory effect of the recombinant protein against *E. coli* K12 was small but statistically significant (Fig. 4B and D). Whilst 6xHis-Hill_BB_C10074 (250 µg/mL) exhibited a highly statistically significant inhibitory effect against *P. aeruginosa* PaO1 (Fig. 4C and D).

### Inhibitory activity of 6xHis-Hill_BB_C10074 against *P. aeruginosa*

**Fig. 5:**
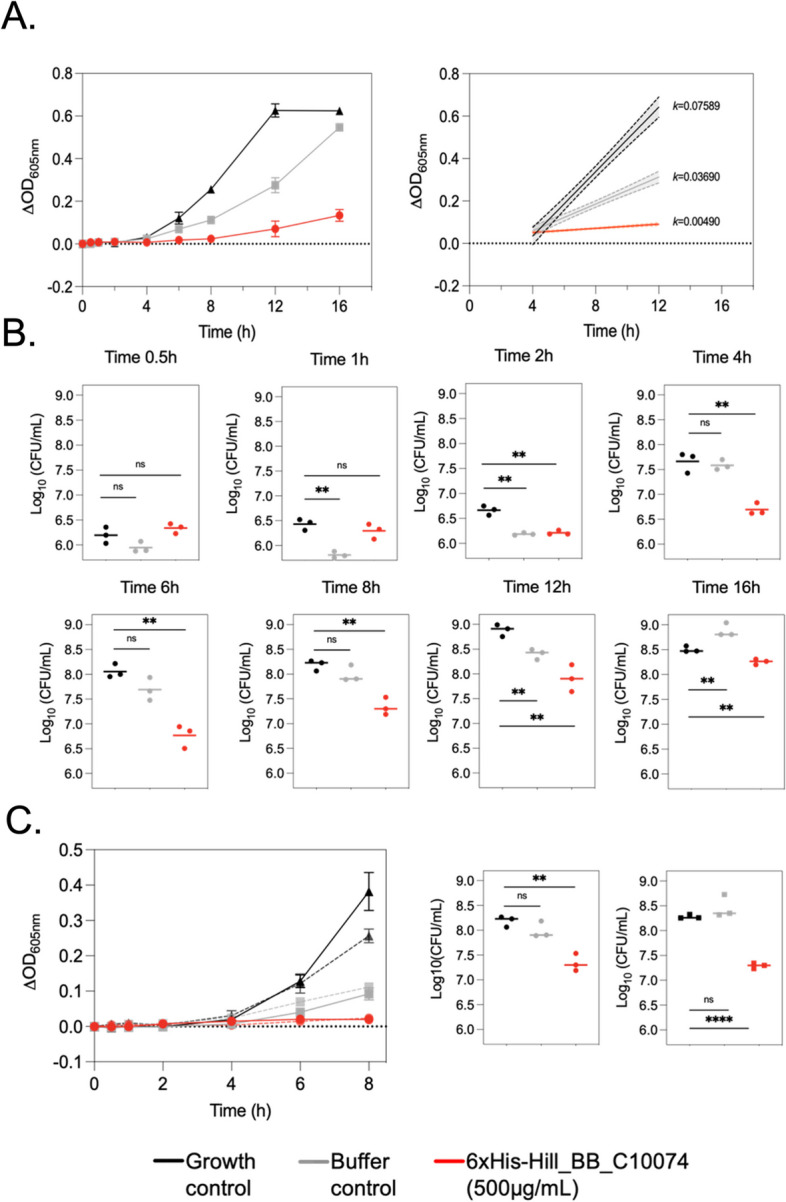
Inhibitory activity of 6xHis-Hill_BB_C10074 against *P. aeruginosa *PaO1. **A **Changes in OD measurements of *P. aeruginosa* PaO1 populations over 16-hours following exposure to either 6xHis-Hill_BB_C10074 (500 µg/mL), Growth control or Buffer control (50mM TrisHCl pH 8.0). Error bars represent standard deviation of the mean. Simple linear regression curves were fitted to the log-growth phase (determined through OD measurements to be between 4–12-hours) to analyse the effect of 6xHis-Hill_BB_C10074 (500 µg/mL) on growth rate during this stage of bacterial growth. Growth rates (*k*) were calculated as the change in OD over time. Error bars represent the 95% CI. **B **The number of CFUs in populations of *P. aeruginosa* PaO1 were calculated periodically over the course of the 16-hour assay.  **C ***P. aeruginosa* PaO1 populations were passaged twice against 6xHis-Hill_BB_C10074 and remained susceptible to the antimicrobial activity of the protein as measured by changes in ODs and the number of viable cells (Log_10_CFU/mL) over 8-hours incubation. All data was inclusive of 3 biological repeats and error bars represent ± standard deviation. Statistics were performed using an unpaired Students’ t-test: ns (not significant) *p* > 0.05, ** *p* ≤ 0.01, **** *p* ≤ 0.0001

The inhibitory activity of 6xHis-Hill_BB_C10074 was determined against *P. aeruginosa*. Assessment of bacterial growth through optical density (OD) measurements showed that 6xHis-Hill_BB_C10074 significantly inhibited the growth of *P. aeruginosa* PaO1 over 16-hours compared with both Growth and Buffer controls (Fig. 5A). The exponential phase growth rate of *P. aeruginosa* PaO1 exposed to 6xHis-Hill_BB_C10074 was 15.5-fold lower than that of the Growth control, and 7.5-fold lower than that of the Buffer control. The antimicrobial phenotype of 6xHis-Hill_BB_C10074 was confirmed through CFU measurements (Fig. 5B). The CFU results showed that both the *P. aeruginosa* PaO1 populations treated with 6xHis-Hill_BB_C10074 and the Buffer control had reduced number of viable cells compared with the Growth control. However, following 2-hours incubation, *P. aeruginosa* PaO1 treated with the Buffer control had a similar number of viable cells to the Growth control population. The CFUs of *P. aeruginosa* PaO1 treated with 6xHis-Hill_BB_C10074 remained significantly reduced compared with the Growth control over the full course of the 16-hour incubation. Bacteria passaged with 6xHis-Hill_BB_C10074 remained susceptible to the inhibitory effect of the protein (Fig. 5C).

**Fig. 6:**
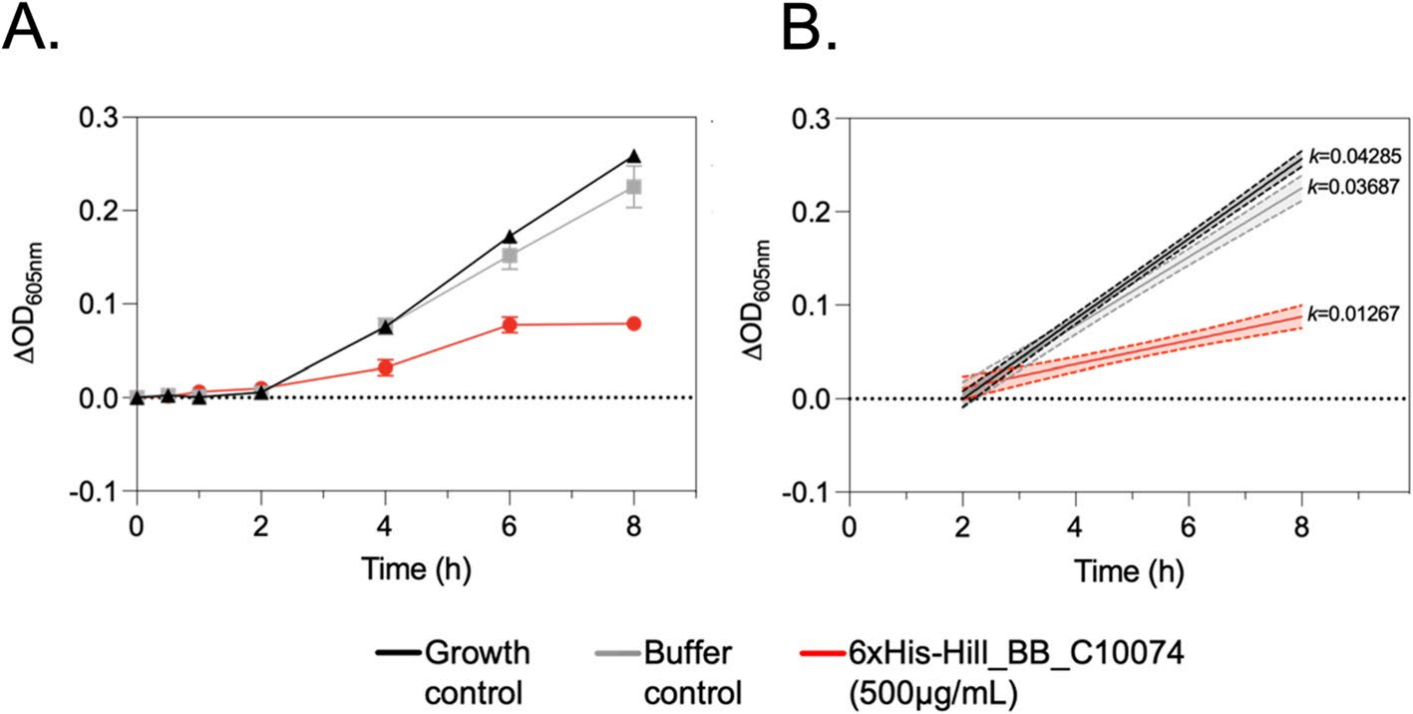
6xHis-Hill_BB_C10074 inhibited the growth of the highly virulent *P. aeruginosa*strain Pa14. **A **Changes in OD measurements over-time of populations of *P. aeruginosa* Pa14 incubated with either 6xHis-Hill_BB_C10074 (500 µg/mL), Growth control or Buffer control (50 mM TrisHCl pH 8.0). Error bars represent ± standard deviation of the mean. **B **Simple linear regression curve fitted to the exponential growth phases of *P. aeruginosa* Pa14 populations between 2–8-hours. Growth rates (*k*) calculated as the change in OD over time. Dotted lines represent ± CIs. All data was inclusive of 3 biological repeats

The inhibitory activity of 6xHis-Hill_BB_C10074 was determined against the highly virulent strain *P. aeruginosa* Pa14. Measurements of OD showed that 6xHis-Hill_BB_C10074 significantly inhibited the growth of *P. aeruginosa* Pa14 over 16-hours compared with both the Buffer and Growth controls (Fig. 6A). The exponential phase growth rate of *P. aeruginosa* Pa14 exposed to 6xHis-Hill_BB_C10074 was 3.4-fold lower than that of the Growth control, and 2.9-fold lower than that of the Buffer control (Fig. 6B).

### Transmission electron microscopy of *P. aeruginosa *PaO1 treated with 6xHis-Hill_BB_C10074

**Fig. 7:**
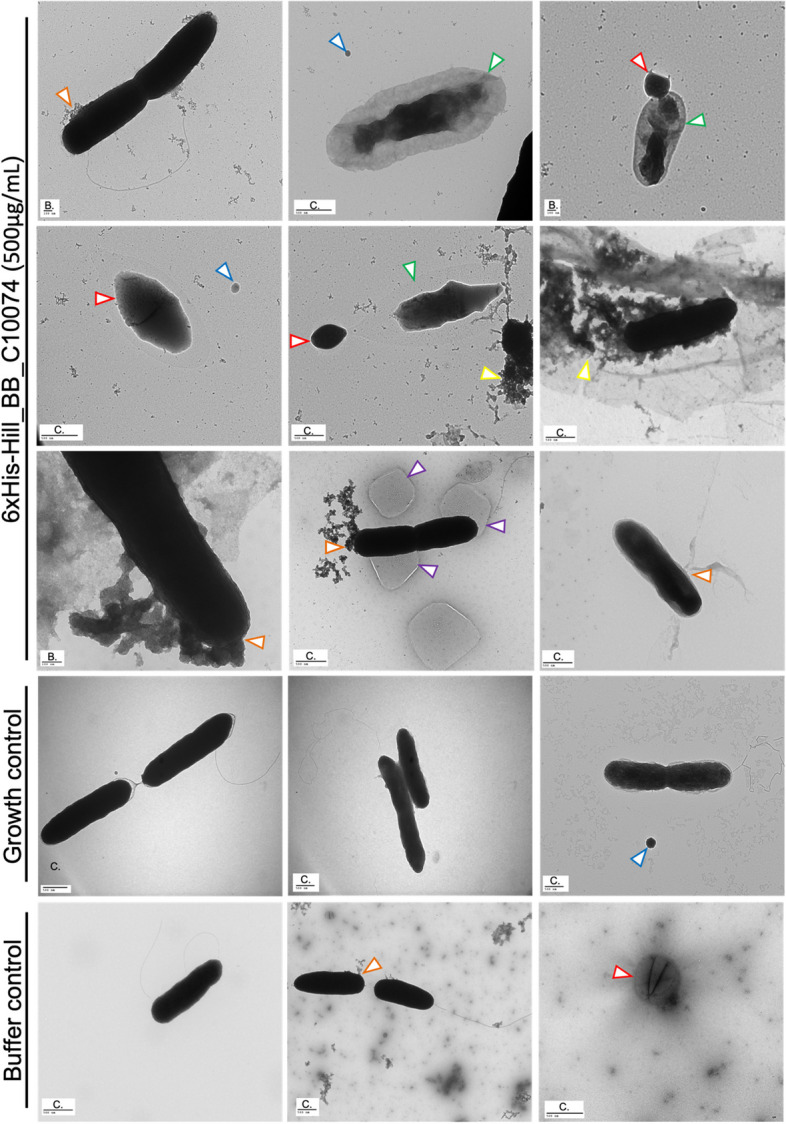
Representative TEM images of *P. aeruginosa *PaO1 incubated with 6xHis-Hill_BB_C10074 and Growth and Buffer controls after 2-hours incubation at 37 °C. TEM indicated that 2-hour exposure to 6xHis-Hill_BB_C10074 (500 µg/mL) affected membrane integrity and induced membrane leakage, formation of small spherical cells and lysis of rod-like cells. Whilst bacteria exposed to the Growth control did not appear to have any membrane damage. Bacterial cells exposed to the Buffer control (50mM TrisHCl pH 8.0) demonstrated some changes in membrane integrity. Blue arrow = vesicles, orange arrow = leakage of intracellular contents, green arrow = lysing cells, yellow arrow = cellular debris, purple arrows = blebbing, red arrow = spherocyte. TEM images shown were selected as representative examples from 3 biological replicates. Scale bars are shown, and letter codes designate the distances of (**A**) as 20 nm, (**B**) as 100 nm, and (**C**) as 500 nm

The effect of 6xHis-Hill_BB_C10074 on *P. aeruginosa* PaO1 was visualised using TEM and compared against the effects of Growth and Buffer controls. Following 2-hours of incubation with 6xHis-Hill_BB_C10074, *P. aeruginosa* PaO1 were leaking intracellular contents (orange arrows) from damaged membranes and cells were surrounded with debris (yellow arrows) (Fig. 7). Vesicles (blue arrows) were present which is consistent with a stress response to membrane acting antimicrobials [28]. The cells were darkly negative stained, possibly from the production of mucin which has previously been considered to enhance the negative staining of *P. aeruginosa* [29]. Some cells had protruding bleb-like structures (purple arrows). Several cells showed densely negatively stained cores with less stained exteriors (green arrows), indicating that the negative stain had entered the cells and indicated that membrane damage had induced lysis. Several small spherical cells were present in *P. aeruginosa* PaO1 populations exposed to 6xHis-Hill_BB_C10074 and Buffer control (red arrows). This morphotype is consistent with spherocytes and is induced by membrane damage [30].

**Fig. 8:**
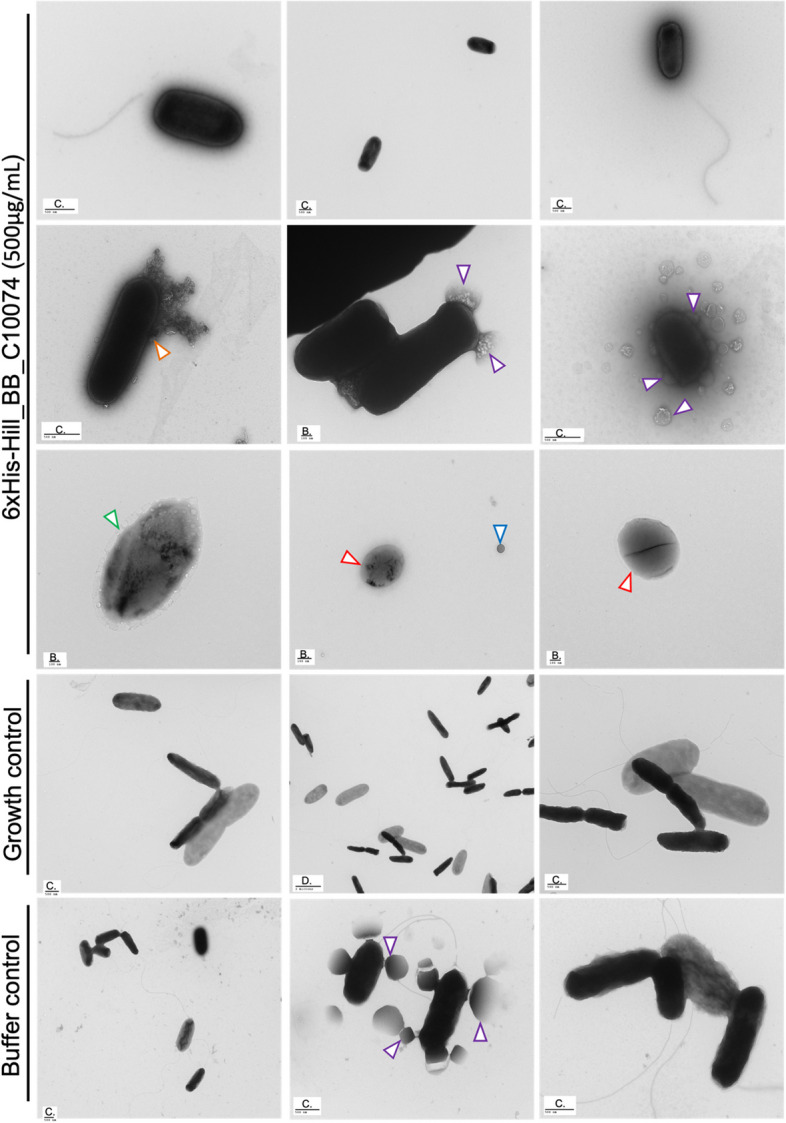
Representative TEM images of *P. aeruginosa *PaO1 incubated with 6xHis-Hill_BB_C10074 and Growth and Buffer controls after 8-hours incubation at 37 °C. TEM showed that 8-hour exposure to 6xHis-Hill_BB_C10074 (500 µg/mL) affected membrane integrity and induced membrane leakage, formation of small spherical cells and lysis of rod-like cells, but some cells appeared healthy and rod-like. There were bacteria in the Growth control population that appeared to be swollen. Some cells in the Buffer control (50mM TrisHCl pH 8.0) were blebbing whilst other cells appeared healthy. Blue arrow = vesicles, orange arrow = leakage of intracellular contents, green arrow = lysing cells, purple arrows = blebbing, red arrow = spherocyte. TEM images shown were selected as representative examples from 3 biological replicates. Scale bars are shown, and letter codes designate the distances of (**A**) as 20 nm, (**B**) as 100 nm, (**C**) as 500 nm, and (**D**) as 2000 nm

Cultures of *P. aeruginosa* PaO1 treated with 6xHis-Hill_BB_C10074 for 8-hours contained a mixture of healthy and damaged cells (Fig. 8). Healthy-looking cells possessed flagella, were not punctured, and were not coated with cellular debris. However, there were many cells that demonstrated either cell surface damage or stress responses such as spherocytes, lysing and blebbing cells. Whilst cells in Buffer and Growth control populations displayed healthier looking phenotypes and appeared to be actively dividing. Several bleb-like structures were still apparent in *P. aeruginosa* PaO1 treated with the Buffer control indicating that the buffer may have some effect on the bacteria.

### Membrane-targeting activity of 6xHis-Hill_BB_C10074 against *P. aeruginosa*

**Fig. 9:**
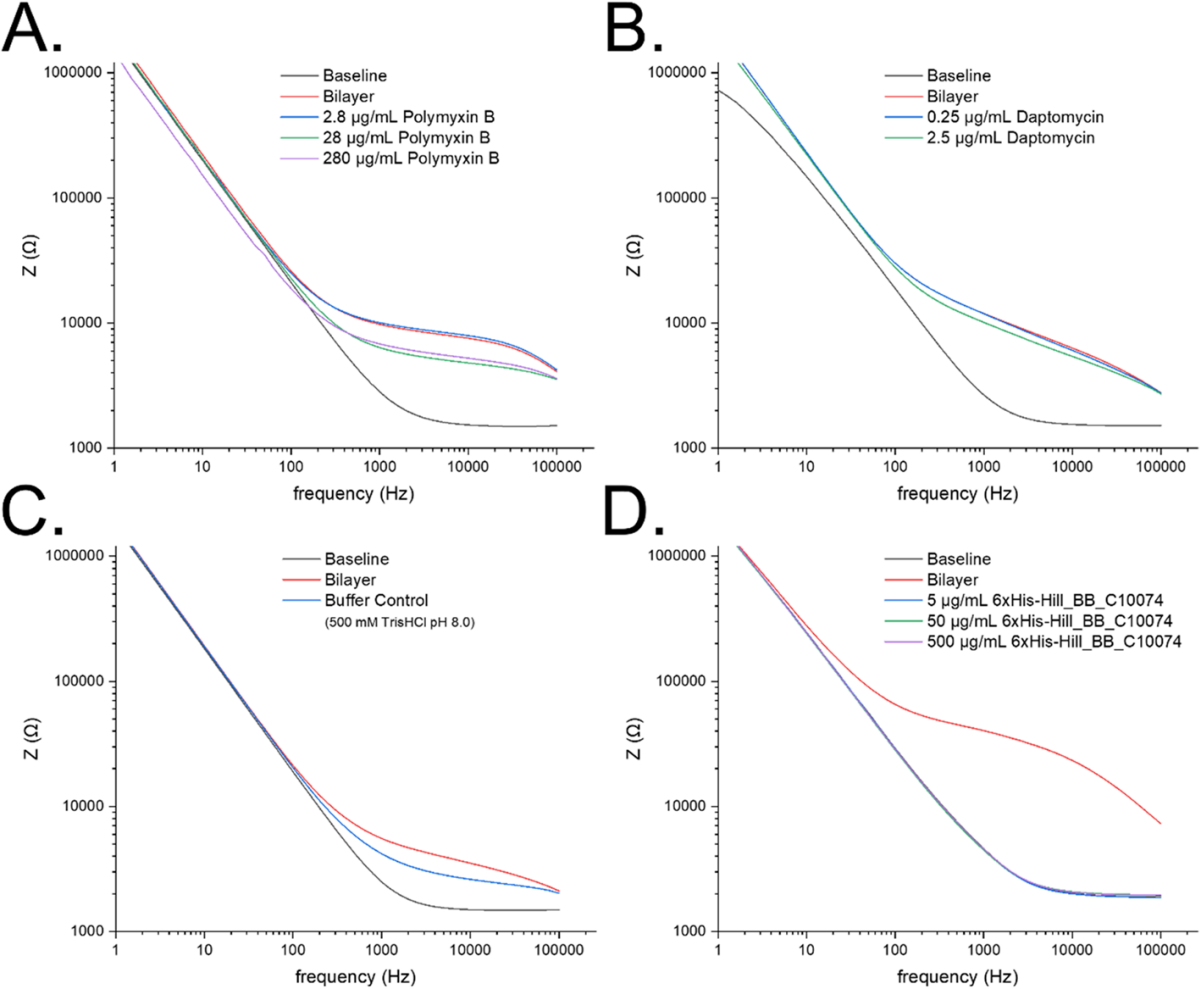
Bode plots of the EIS of SLBs formed from *P. aeruginosa *PaO1 OMVs. EIS measured the impedance of electrolytes across a SLB built from *P. aeruginosa* PaO1 OMVs. SLBs treated with polymyxin B **A **at 280 µg/ml, 28 µg/ml and 2.8 µg/ml, showed that (from the concentrations tested) a concentration of 28 µg/ml is required to reduce the impedance of the SLB. SLBs treated with daptomycin **B **do not reduce impedance as the drug requires binding to teichoic acids which are specific to Gram-positive bacterial membranes. SLBs treated with the Buffer control (500 mM TrisHCl pH 8.0) **C **showed a slight reduction in impedance, indicating that high concentrations of TrisHCl affected membrane integrity. SLBs treated with 6xHis-Hill_BB_C10074 **D **demonstrated that even at the lowest concentration tested of 5 µg/mL, membranes provided no impedance to the electrolyte showing that the membrane was destroyed. This low concentration (5 µg/mL) was 1000-fold lower than the concentration used to successfully inhibit bacteria in whole-cell assays and contained just 0.5 mM Tris. All data was obtained using the Nova software. EIS data are representative of 4 independent electrodes

To determine whether 6xHis-Hill_BB_C10074 targeted the *P. aeruginosa* outer membrane, Electrochemical impedance spectroscopy (EIS) of supported lipid bilayers (SLBs) formed of *P. aeruginosa* PaO1 OMVs, was performed to measure the interaction between 6xHis-Hill_BB_C10074 and the membrane. SLBs were formed on the poly(3,4-ethylenedioxythiophene) polystyrene sulfonate (PEDOT:PSS) coated gold electrodes to electrically monitor the resistance of the bilayer. SLBs treated with the previously established positive control of Gram-negative membrane targeting polymyxin B were damaged in a dose-dependent manner, where increasing concentrations of polymyxin B exhibit a decrease in membrane resistance and a trend towards the device baseline (Fig. 9A) [31, 32]. Whilst SLBs treated with the negative control of daptomycin, which only functions against Gram-positive bacterial membranes, were not affected (Fig. 9B). Results showed that the Buffer control slightly reduced the integrity of the SLB indicating that the buffer damaged the *P. aeruginosa* PaO1 membrane (Fig. 9C.). However, this was at a concentration 10X greater than that used in whole-cell assays. Importantly, SLBs treated with 6xHis-Hill_BB_C10074 were completely disrupted at every concentration tested (500 µg/mL – 5 µg/mL) (Fig. 9D.). This confirmed the potent membrane-disrupting efficacy of 6xHis-Hill_BB_C10074.

## Discussion

To meet the challenge of AMR, development of clinically relevant novel antimicrobials is urgently required. AMPs are a promising class of novel therapy due to their broad spectrum of activity, rapid antimicrobial action and their resulting low propensity to induce resistance.

Attacins, a family of AMPs, that have been solely identified in insects represent one of the largest classes of discovered AMPs and are thought to be selective towards Gram-negative bacteria. The attacin Hill_BB_C10074 that was first identified in larvae that had been fed a plant oil-based diet [21]. In this study, recombinant Hill_BB_C10074 was found to possess potent antimicrobial activity against the bacterial pathogen, *P. aeruginosa*. As a member of the ESKAPE (*Enterococcus faecium*, *S. aureus*, *Klebsiella pneumoniae*, *Acinetobacter baumannii*, *P. aeruginosa* and *Enterobacter* spp.) group of pathogens, *P. aeruginosa* is now recognised as a species of bacteria with critical priority for the development of new antimicrobial therapies. Prevalence of *P. aeruginosa* infections has been increasing and is now associated with ~ 7% of all nosocomial infections [33]. The pathogen is particularly responsible for infections in intensive care units (ICU) with isolates of *P. aeruginosa* identified in 23% of all ICU infections and mortalities of ventilator associated infections secondary to *P. aeruginosa* are as high as 42.8% [34]. *P. aeruginosa* is also a major causative organism in lung and soft tissue infections in cystic fibrosis and burns patients [33].

Attacins have been described for their antimicrobial selectivity for Gram-negative bacteria. In line with the attacin family, Hill_BB_C10074, selectively inhibited the growth of the Gram-negative bacteria *E. coli* and *P. aeruginosa*, whilst it did not alter the growth of the Gram-positive species tested, *S. aureus*. The antimicrobial action of Hill_BB_C10074 was most pronounced against *P. aeruginosa*, and the attacin was shown to significantly inhibit the growth of the bacteria for over 16-hours. Furthermore, when populations of bacteria were subsequently re-exposed to Hill_BB_C10074 they remained susceptible to its antimicrobial action demonstrating that there was no resistance development, as expected for AMPs that target bacterial membranes. TEM imaging showed that Hill_BB_C10074 caused *P. aeruginosa* cells to leak intracellular contents and induced lysis. Several other cellular phenotypes such as blebbing and spherocyte formation were demonstrated in response to Hill_BB_C10074. *P. aeruginosa* has been shown to rapidly transition into spherocytes in response to membrane acting antimicrobials, indicating that the attacin may function against the outer membrane of the bacterium. Spherocytes possess disrupted outer membranes however remain viable and can transition back to rod-shaped cells following removal of the antimicrobial [30]. The outer membrane activity of the attacin, Hill_BB_C10074, was confirmed through EIS of SLBs formed of *P. aeruginosa* membranes. Even at the lowest concentration tested (5 µg/mL) Hill_BB_C10074 completely disrupted the *P. aeruginosa* membrane. Notably, Hill_BB_C10074 (5 µg/ml) possessed a greater membrane disruptive power than the membrane-targeting clinical antibiotic polymyxin B which has been shown to have an minimum inhibitory concentration of 64 µg/ml [35]. Hill_BB_C10074 also inhibited the growth of the highly virulent clinical strain *P. aeruginosa* Pa14 that was first isolated from an infected wound of a burn patient [36].

There has been a recent rise in the use of artificial intelligence to aid drug development projects [37]. *In silico* tools such as AF2 have increased accessibility to the field of AMP discovery, allowing for rapid and cheap prediction of novel AMP candidates. Several AMPs have recently been realised through predictive softwares that performed rational design of candidate sequences [38, 39]. Predictive tools can also be used to identify the functionality of AMPs through inferred SARs. We employed the AF2 and Dali homology severs to predict that the attacin, Hill_BB_C10074, formed a multimeric unit, likely a homotrimer, that shared structural space with Gram-negative bacterial porins. From this structure prediction, we hypothesised that Hill_BB_C10074 disrupted the Gram-negative outer membrane through pore formation. These predictions were supported in vitro through EIS of attacin-treated *P. aeruginosa* membranes that confirmed that Hill_BB_C10074 damaged the bacterial outer membrane. The combination of predictive tools with in vitro phenotypic testing has helped characterise Hill_BB_C10074 as a potent membrane acting antimicrobial.

## Conclusions

In this study, an attacin from *H. illucens* was found to possess potent antimicrobial activity against the serious pathogen *P. aeruginosa*. *In silico* tools predicted that Hill_BB_C10074 formed a homotrimer and structure-activity-relationship inferred that the protein formed pores in the outer membrane of Gram-negative bacteria. We demonstrated that Hill_BB_C10074 can disrupt the outer membrane of *P. aeruginosa* PaO1. Hill_BB_C10074 inflicted greater damage to the *P. aeruginosa* membrane than the current antibiotic used in the clinic as a last line of defence, polymyxin B. Our work shows that Hill_BB_C10074 is a promising candidate for future development of antimicrobials to treat Gram-negative bacterial infections, particularly those caused by *P. aeruginosa.*

## Materials and methods

### Computational characterisation of Hill_BB_C10074

The Hill_BB_C10074 signal peptide was identified using SignalP-5.0 [40]. Tertiary structure of the mature Hill_BB_C10074 was modelled using the software AF2 [41] generating Protein Databank (PDB) co-ordinate files that were visualised using the ChimeraX [42] environment. Electrostatic and hydrophobic surfaces of the AF2 prediction were modelled using the in-built features of ChimeraX. Structural homologies were searched for using the Dali server [43] which scanned the co-ordinate files against the entire PDB database.

### Expression and purification of recombinant 6xHis-Hill_BB_C10074

Codon-optimised mature Hill_BB_C10074 DNA was synthesised by GeneArt (ThermoFisher) and subsequently subcloned into pET28a(+), using the BamHI and NotI restriction enzyme sites, which provided an N-terminal 6xHis tag, T7 tag and thrombin cleavage site. The plasmid was freshly transformed into *E. coli* BL21(DE3) cells. Bacteria harbouring the plasmid were selected for through their kanamycin antibiotic resistance. Cultures of transformed bacteria were grown in baffled flasks at 37 °C, shaking at 220 rpm, until OD_600nm_ 0.4–0.7 was reached. Protein expression was induced with isopropyl-β-D-thiogalactopyranoside (IPTG) at 37 °C, shaking at 220 rpm, for 4-hours. The bacteria were sonicated (5-minutes total processing, 25 kHz) in ice-cold lysis buffer (50 mM TrisHCl, pH 7.0, 150 mM NaCl and, 1.5X cOmplete^™^ protease inhibitor cocktail). Inclusion bodies were isolated from the lysate through a series of centrifugations all performed at 10,000 x*g* for 20-minutes at 4 °C. Initially, pellets were resuspended in a buffer of 100 mM TrisHCl pH 7.0 supplemented with 10 mM EDTA, 10 mM DTT and, 0.5% v/v Triton X-100, pH 7.0 and centrifuged. Membranes and membrane proteins were solubilised from the pellet through suspension and centrifugation in a buffer of 100 mM TrisHCl supplemented with 5 mM EDTA, 5 mM DTT and, 2% v/v Triton X-100, pH 7.0. Nucleic acids were removed from the pellet through suspension and centrifugation in a buffer of 100 mM TrisHCl supplemented with 5 mM EDTA, 5 mM DTT and, 1 M NaCl, pH 7.0. Isolated inclusion bodies were solubilised in a buffer of 50 mM TrisHCl, 8 M urea, 500 mM NaCl, 5 mM imidazole and 5 mM DTT pH 8.0. Recombinant 6xHis-Hill_BB_C10074 was batch purified through nickel-affinity chromatography. Solubilised inclusion bodies were applied *via* syringe to a HisTrap^™^ HP column (Cytiva LifeSciences) at a flow rate of 5 mL/min. Flow through was discarded and the column was washed with 10 column volumes of solubilisation buffer. Proteins were eluted using a buffer composed of solubilisation buffer and a gradient imidazole concentration (50 mM – 400 mM). Eluted proteins were dialysed against a buffer containing 750 mM arginine, 500 mM urea, 5 mM CaCl_2_, 1 mM oxidised glutathione, 3 mM reduced glutathione, pH 8.0. Dialysed recombinant 6xHis-Hill_BB_C10074 was exchanged into 20 mM TrisHCl pH 8.0 buffer using PD10 desalting columns. Recombinant 6xHis-Hill_BB_C10074 was concentrated by lyophilisation. A quality control was included that ran blank buffers through the purification process, this measured the effect of the final buffer preparation in down-stream assays and is referred to as “Buffer control”.

### Phase-contrast microscopy

To visualise inclusion bodies, intact *E. coli* cells expressing 6xHis-Hill_BB_C10074 were visualised through phase contrast microscopy using an Olympus BX53 microscope (x100 magnification).

### SDS-PAGE and western blotting

Total, soluble and insoluble protein fractions of whole-cell lysates were analysed through SDS-PAGE and western blotting. Insoluble proteins were fractionated from soluble proteins through centrifugation at 16,000 *xg* for 1-minute. Total protein and fractionated samples were resuspended in 1X SDS sample buffer supplemented with 1X reducing agent and heated at 85 °C for 5-minutes. Samples were loaded onto an 4–12% BisTris gel and resolved through electrophoresis. Proteins separated by SDS-PAGE were visualised with Coomassie Blue stain. Western blotting confirmed the presence of 6xHis-Hill_BB_C10074. Proteins were transferred from SDS-PAGE onto a PVDF membrane (25 V, 1.3 A, 5-minutes). Membranes were blocked in a solution of 5% w/v BSA. Rabbit polyclonal primary antibody (0.5 µg/mL, ab14923) bound the 6xHis tag of 6xHis-Hill_BB_C10074 and an HRP-conjugated goat anti-rabbit secondary antibody (1:5,000 dilution, ab97051) detected the primary antibody. HRP was probed for using chemiluminescent substrate (Supersignal PLUS West Pico) and visualised.

### In vitro antimicrobial activity assay

To determine the antimicrobial activity of 6xHis-Hill_BB_C10074 against *E. coli* K12, *S. aureus* NCTC 10,788, and *P. aeruginosa* strains PaO1 and Pa14 antimicrobial screens were performed using a 96-well plate format that monitored the growth of bacteria through OD and CFU measurements. Briefly, 50 µL of overnight bacterial cultures that had been diluted 1:100 into fresh Mueller Hinton (MH) broth were used to inoculate wells containing 6xHis-Hill_BB_C10074 (either 250 µg/mL or 500 µg/mL depending on batch-to-batch variation in recombinant protein purity) suspended in MH broth to a total well volume of 200 µL. OD_605nm_ (Absorbance 96, Byony) and CFU measurements of wells were measured periodically. Controls were included that measured the undisturbed growth of bacteria in MH broth and the growth of bacteria in the presence of the proteins’ buffer, these controls are termed “Growth” and “Buffer” control, respectively. Buffer control was either 20 mM or 50 mM TrisHCl pH 8.0 for experiments studying 6xHis-Hill_BB_C10074 at concentrations 250 µg/mL or 500 µg/mL, respectively.

### Transmission electron microscopy

Volumes of 400 µL of *P. aeruginosa* PaO1 culture exposed to 6xHis-Hill_BB_C10074 (500 µg/mL) at 37 °C for 0-hours, 2-hours and 8-hours were fixed in 200 µL 8% v/v paraformaldehyde (PFA) in PBS pH 7.4 at room temperature for 45-minutes. Suspensions were centrifuged (15-minutes at 4,000 x*g*) and pellets were washed twice in 200 µL 1X PBS pH 7.4 and then resuspended to a final volume of 20 µL in 1X PBS pH 7.4. A volume of 5 mL of fixed cells were adsorbed for 30-seconds onto a glow discharged 400-mesh copper grid with carbon film attached. Grids were washed twice for 30-seconds with nuclease free H_2_O (Invitrogen) and stained for 30-seconds with 1% w/v uranyl acetate. Grids were viewed using a Tecani G2 Transmission Electron Microscope (FEI) at 200 keV.

### Electrochemical impedance spectroscopy

Outer membrane vesicles (OMVs) of *P. aeruginosa* PaO1 bacteria were isolated from fresh cultures of *P. aeruginosa* PaO1 that had been grown in baffled flasks at 37 °C with shaking at 220 rpm until OD_600nm_ 1.5 [44]. Bacteria were centrifuged (6,000 x*g*, 5-minutes, 4 °C) and the supernatant was sequentially passed through 0.45µM then 0.22 µM filters. OMVs were isolated by ultracentrifugation at 140,000 *xg* for 3-hours at 4 °C. Collected pellets were washed in PBS supplemented with 2 mM MgCl_2_ at 16,000 x*g* for 30-minutes at 4 °C. Final pellets were resuspended in the buffer of PBS supplemented with 2 mM MgCl_2_, aliquoted, and stored at -80 °C before use in the supported lipid bilayer (SLB).

Microfabricated electrode devices were fabricated in-house. Negative photoresist (AZ^®^ nLOF 2035) was spin coated onto wafers (45-seconds at 3,000 rpm; Laurell WS-650-Mz-23NPPB Spin Processor), baked at 110°C for 1-minute and briefly exposed to UV. Glass 4” wafers that had been cleaned and baked for 3-minutes at 110 °C were developed (AZ^®^ 726 MIF). To deposit the electrodes and interconnects, wafers were treated with 30% oxygen plasma (1-minute, Diener Electronic FEMTO), and then underwent e-beam evaporation of a 10 nm titanium adhesion layer followed by 100 nm gold (Kurt J Lesker PVD-75). Lift-off performed for 30-minutes in acetone. For parylene-C deposition, wafers were exposed to 30% oxygen plasma (1-minute) and briefly soaked in 3% w/v silane A174, [3-(Methacryloyloxy)propyl]trimethoxysilane. Wafers were dried and a 2 μm layer (approx.) of parylene-C was deposited (PDS 2010 Labcoter 2). An anti-adhesive layer of 2% v/v Micro-90^®^ solution was spin coated then 2 μm parylene-C was deposited. AZ^®^ 10XT positive photoresist was subsequently deposited, exposed to UV before development in AZ^®^ 726 MIF. Electrodes and contact pads were exposed by reactive-ion etching (Oxford 80 PlasmaLab Plus). A PEDOT:PSS mixture was prepared of 94% v/v Clevios PH 1000, 4.75% v/v ethylene glycol, 1% w/v (3-glycidyloxypropyl)trimethoxysilane and 0.25% v/v 4-dodecylbenzenesulfonic acid and homogenised in a bath sonicator (30-minutes, 40 kHz, 100 W). Device substrates were plasma treated for 2-minutes then spin coated with the PEDOT:PSS mixture (45-seconds at 3,000 rpm; Laurell WS-650-Mz-23NPPB Spin Processor) and baked (90 °C, 1-minute). Contact pads were scrubbed with water to remove excess PEDOT:PSS, and the parylene-C sacrifical layer peeled off. Devices were baked at 140 °C for 1-hour to cross-link. Glass wells were attached to the device substrate using polydimethylsiloxane and baked (90 °C, 1-hour). For SLB formation, devices were surface treated with the *P. aeruginosa* OMVs (100 µl per device). Residual excess OMVs were washed from the wells with PBS. Adsorbed OMVs were ruptured onto the surface of the well following exposure to Dipalmitoulphosphatidylcholine – Dioleoyl-3-trimethylammonium propane liposomes (100 µl, 4 mg/ml) [45, 46].

SLBs were exposed to varying concentrations of 6xHis-Hill_BB_C10074 (5–500 µg/mL), the Buffer control (500mM Tris pH 8.0), daptomycin (0.25–2.5 µg/mL, supplemented with 3 mM CaCl_2_ co-factor) or polymyxin B (2.8–280 µg/mL). Concentration ranges of antibiotic controls were selected to encompass clinical relevance [35, 47]. SLBs were exposed for 20-minutes then washed in the PBS buffer supplemented with the electrolyte 2 mM MgCl_2_. EIS was performed by applying a sinusoidal voltage to the SLB, inducing the flux of ions from the electrolyte that were subsequently transduced onto the conducting polymer and measured by potentiostat (AutoLab PGSTAT204 Metrohm) [45].

### Data and statistical analysis

All statistical analyses and graphical representations were performed using GraphPad Prism, version 9 (GraphPad Software, San Diego, California USA, www.graphpad.com).

### Supplementary Information

**Additional file 1: Supplementary Figure 1. **Full length gels and blot images. Uncropped raw images of the gels and blots shown in Fig. 3. Cropped regions of the images are boxed with the dashed line.

## Data Availability

All data generated and analysed during this study are included in this published article. The datasets used and/or analysed during the current study are available from the corresponding author on reasonable request.

## Abbreviations

AMR: Antimicrobial resistance
AMPs: Antimicrobial peptides
CAMPR3: Collection of Antimicrobial Peptides R3 database
AF2: AlphaFold v2.1.0
>SAR: Structure-Activity-Relationship
EIS: Electrochemical impedance spectroscopy
SLBs: supported lipid bilayers
IPTG: isopropyl-β-D-thiogalactopyranoside
OMVs: outer membrane vesicles
PEDOT-PSS: poly(3,4-ethylenedioxythiophene) polystyrene sulfonate
