# Supplementary material for: An attacin antimicrobial peptide, Hill_BB_C10074, from Hermetia illucens with anti-Pseudomonas aeruginosa activity
Source: BMC Microbiol. 2023 Dec 1;23:378. doi: 10.1186/s12866-023-03131-1 (PMC10690985; doi:10.1186/s12866-023-03131-1)
Supplement: Supplementary file 1 — Additional file 1: Supplementary Figure 1. Full length gels and blot images. Uncropped raw images of the gels and blots shown in Fig. 3. Cropped regions of the images are boxed with the dashed line. [file 12866_2023_3131_MOESM1_ESM.docx]

**Supplementary Information**

**
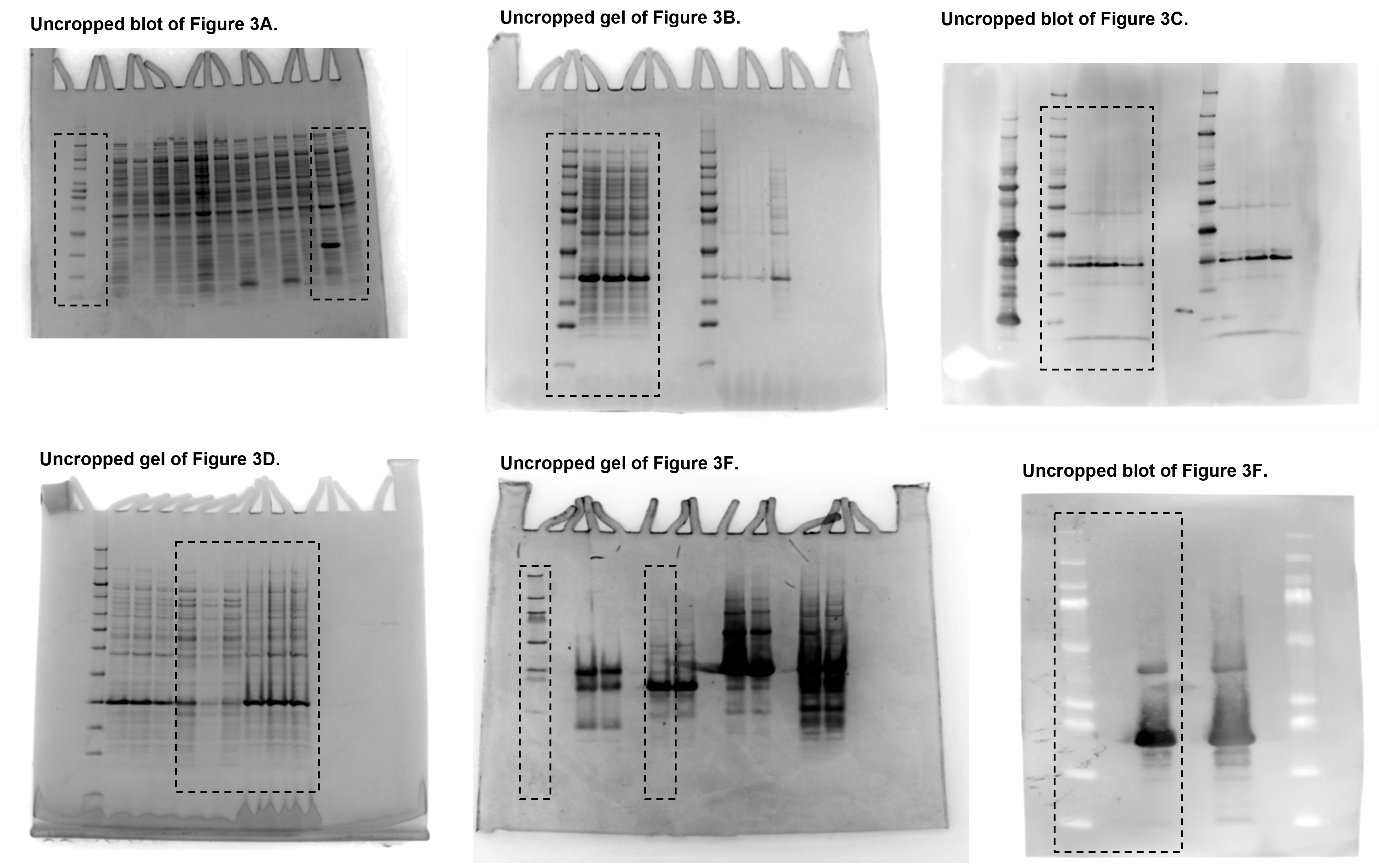
**

**Supplementary Figure 1. Full length gels and blot images.** Uncropped raw images of the gels and blots shown in Figure 3. Cropped regions of the images are boxed with the dashed line.
